# Approximating Community Water System Service Areas to Explore the Demographics of SDWA Compliance in Virginia

**DOI:** 10.3390/ijerph182413254

**Published:** 2021-12-16

**Authors:** Cristina Marcillo, Leigh-Anne Krometis, Justin Krometis

**Affiliations:** 1 Department of Biological Systems Engineering, Virginia Polytechnic Institute and State University, Blacksburg, VA 24061, USA; 2 Advanced Research Computing, Virginia Polytechnic Institute and State University, Blacksburg, VA 24061, USA; jkrometis@vt.edu

**Keywords:** drinking water, infrastructure, Safe Drinking Water Act, environmental justice, community water system, water quality

## Abstract

Although the United States Safe Drinking Water Act (SDWA) theoretically ensures drinking water quality, recent studies have questioned the reliability and equity associated with community water system (CWS) service. This study aimed to identify SDWA violation differences (i.e., monitoring and reporting (MR) and health-based (HB)) between Virginia CWSs given associated service demographics, rurality, and system characteristics. A novel geospatial methodology delineated CWS service areas at the zip code scale to connect 2000 US Census demographics with 2006–2016 SDWA violations, with significant associations determined via negative binomial regression. The proportion of Black Americans within a service area was positively associated with the likelihood of HB violations. This effort supports the need for further investigation of racial and socioeconomic disparities in access to safe drinking water within the United States in particular and offers a geospatial strategy to explore demographics in other settings where data on infrastructure extents are limited. Further interdisciplinary efforts at multiple scales are necessary to identify the entwined causes for differential risks in adverse drinking water quality exposures and would be substantially strengthened by the mapping of official CWS service boundaries.

## 1. Introduction

Previous research has reported economic and racial/ethnic disparities in access to centralized, treated drinking water [1,2]. Although infrastructure investments have expanded access to community water systems, the water provided is not always of sufficient quality and quantity to meet household needs. Over the last decade, several environmental justice studies have investigated the potential association between community demographics on the quality, access, and affordability of water provided by public and community water systems and their compliance with the Safe Drinking Water Act (SDWA) (summarized in Table S1). Recent literature has suggested that communities that are less affluent [3,4,5] or have larger Black or Hispanic populations [6,7,8] have poorer in-home water quality or are served by systems with more SDWA violations; only one identified study examining the Revised Arsenic Rule in Arizona failed to find a significant link between sociodemographic indicators and water quality [9]. Direct comparison between studies is difficult, as results are dependent on geographic unit, system sizes included, health-based (HB) targets investigated, and demographic variables used in the specific statistical models employed. Although most previous work has examined the influence of potential predictors individually, recent national assessments appear to indicate that predictive factors can be interrelated. Switzer and Teodoro [7] demonstrated that when poverty level exceeded 30% in a county, the likelihood of an HB drinking water violation increased in CWSs that served primarily Hispanic and Black populations. Similarly, Allaire et al. [3] found that low-income, minority populations were served by systems with a higher likelihood of total coliform violations.

One major criticism of previous work has been the various geospatial methodologies used to delineate CWS service areas and summarize demographic characteristics [10,11]. In the online Safe Drinking Water Information System (i.e., SDWIS, the electronic public record maintained by the USEPA), water systems report service areas and populations served at the county or equivalent level. While many larger CWSs (>10,000 served) utilize geographic information system technology that allows for real-time integration of infrastructure and service updates at a fine scale, most smaller-sized CWSs (<3300 served) rely on outdated and at times even paper maps to track infrastructure and service area changes, as detailed digital mapping can be cost prohibitive [12]. For this reason, many studies [3,4,6,7,13] have used “county” as their geographic unit of analysis and have excluded certain systems sizes, such as very small (<500 served) and small (501–3300 served), from their analysis. While it is true that medium, large, and very large CWSs serve 91% of the population, small and very small systems comprise 81% and 55% of national PWSs and CWSs, respectively [14]. There is also increasing evidence that smaller systems incur a greater number of SDWA violations [3,15,16]. Few studies have attempted to outline service areas at geographic unit smaller than the county or independent city scale, which is a necessary step before consumer exposure at the tap can be quantified. To the authors’ knowledge, CalEnviroScreen, a mapping tool created by the California Office of Environmental Health Hazard Assessment, is currently the only publicly available statewide assessment of consumer drinking water exposure at a fine-scale level (i.e., census tract [17]).

The broad goal of this study is to apply a novel geospatial method to delineate system service areas at the zip code scale in ESRI’s ArcGIS Pro for previously geocoded Virginia CWSs [15] in order to explore the potential impacts of demographics at a finer scale than county-level. Negative binomial regression is used to identify potential relationships between CWS violations (2006–2016) and service area demographics (race/ethnicity, homeownership, population over 65 years of age), rurality (rural vs. urban), and system characteristics (size, ownership, source water type). This exercise allowed for the explicit examination of the research question: Are certain types of SDWA violations (i.e., monitoring and reporting (MR), health based (HB)) more likely in communities with low homeownership rates, a high proportion of racial and ethnic minorities, and/or rural areas? While the results presented here are specific to Virginia, this methodology, which uses only publicly available data, has the potential for national application within the United States and/or application to explorations of community impacts on infrastructure extents in other settings where formal geospatial data are limited.

## 2. Materials and Methods

### 2.1. Geocoded Community Water System Data

This study focused on a previously geocoded dataset of 671 CWS in Virginia [15], which represent those systems that could be geocoded to the zip code level using ESRI’s ArcGIS Pro (Environmental Systems Research Institute, Redlands, CA, USA). Note that while a public water system (PWS) maintains at least 15 service connections or serves at least 25 people for at least 60 days/year, a community water system (CWS) is a PWS that serves the same population year-round. Comparison analyses indicated that systems that could not be geocoded were generally smaller-sized and dependent on groundwater, though there were not significant differences between these systems and those that could be geocoded. Geocoded systems serve over 5.3 million Virginians, or approximately 75% of the total state population dependent on community water supplies. Noncommunity transient and noncommunity nontransient water systems were excluded from study. Due to the method of CWS service area delineation described in subsequent sections, the final dataset analyzed in this study includes 662 CWSs. CWS characteristics (such as source water type, owner, violations, and population served determining system size) were obtained from the EPA’s Federal SDWIS for 2006–2016. Systems were categorized into sizes based on the following EPA guidelines: very small (<500 served), small (501–3300 served), medium (3301–10,000 served), large (10,001–100,000 served), and very large (>100,000 served). Population estimates obtained from SDWIS inherently exclude residents served by alternate sources (i.e., private wells). Violation types were divided into two separate categories: MR and HB. HB violations include all maximum contaminant level, maximum residual disinfection levels, and treatment technique violations. MR violations designate a CWS failure to monitor treatment, test water quality, and/or report results to the primacy agency. Violation counts were summed over each study year and normalized by the number of active years for each CWS, to address artificially low violation sums associated with inactivated systems.

### 2.2. Service Area Delineation

**Figure 1:**
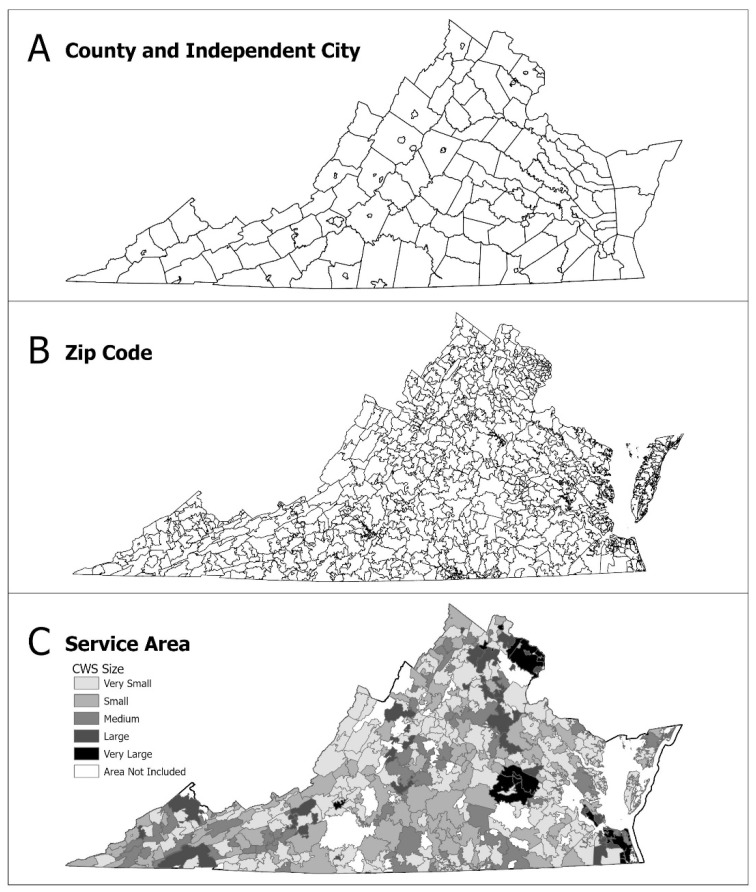
Comparison of Virginia (**A**) county and independent cities (*n* = 134), (**B**) zip codes (*n* = 886), and (**C**) this study’s results for community water system (CWS) service areas (*n* = 662) at the zip code scale by CWS size. Note: Some service areas overlap, obscuring a full view of all delineated service areas. Geographies not included in a service area do not imply that there is no service from CWSs but were excluded due to systems that previously could not be geocoded. All geographies are from the 2000 US Census Tiger shapefiles.

For the purposes of this study, populations were assigned to CWSs based on proximity, i.e., systems were assumed to serve communities closest to the treatment plant. Service area delineation was automated in ArcGIS Pro using a Python script developed with the ArcPy package in Jupyter Notebook. Any CWS with a reported zero population served in EPA’s SDWIS was removed from the study dataset (i.e., these are most likely systems that provide treated source water to multiple smaller systems). Zip codes that did not fit hierarchically within county boundaries were joined to a county based on the zip code’s center of area. The overwhelming majority (90.6%) of zip codes had at least 70% of their area in the county to which they were joined, supporting this as a reasonable approximation. Based on the authors’ prior study [15], 40% of VA CWSs could not be geocoded and were therefore excluded from service area delineation. The population served by non-geocoded systems was removed from analysis as follows: first, using SDWIS information on population served by county, a percentage of population served by geocoded versus non-geocoded CWSs was calculated for each county. The population in each zip code was adjusted to reflect only the percentage of the population served by geocoded CWSs in the county to which it was joined. As the SWDIS population estimates inherently exclude residents served by alternate sources (i.e., private wells), this adjustment also works to exclude those same populations from the following steps. CWS service areas were then created iteratively. Starting with the geocoded CWS with the smallest population served according to SDWIS, a Near Table was generated that ranks zip codes closest to the CWS. Using this ranking, zip codes were cycled through and marked as “served” by the selected CWS, until the population that the selected CWS served was completely attributed to zip codes or there were no more zip codes left in the CWS’s respective county. A new shapefile layer was created for each service area. Additionally, a small number of CWSs (i.e., independent cities with very small geographies) could not be delineated at the zip code scale via this method. Overall, service areas for 662 CWSs were delineated across Virginia out of 1133 total systems. A detailed diagram of this iterative procedure for service area delineation is provided in Figure S1. Results of CWS service area delineation at the zip code scale are compared to the county and independent city scale, which is the unit of analysis in the majority of previous similar studies examining demographics and SDWA violations (Figure 1; [3,4,6,7,13]).

### 2.3. Demographic Variables

In keeping with previous research [15], this study relied upon the USDA’s rural urban commuting area (RUCA) codes to define rurality, as translated to the zip code unit by The University of Washington [18]. Each Virginia zip code corresponds to the following RUCA category: urban core (RUCA = 1), urban (2 and 3), large town core (4), large town (5 and 6), small town core (7), small town (8 and 9), and isolated rural area (10). Census demographics for zip codes were obtained from the 2000 decennial Census [19] and include total population, race, ethnicity, population that is 65 years of age and older (i.e., a group uniquely vulnerable to many environmental health hazards as immune function and host defenses decline; [20]), and homeownership, which is an established socioeconomic status (SES) proxy [21,22]. Demographic factors available at the zip code scale were limited; for example, education and median household income were not available at the zip code scale for the 2000 Census, and even fewer demographic variables were available for the 2010 Census at this same scale. Therefore, the 2000 decennial Census was selected as the best available demographic data for the targeted geographic unit of interest. Descriptive statistics of VA demographics, system characteristics, and RUCA codes are presented in Tables S2 and S3. Demographic variable values for each CWS service area were estimated using an area weighted mean.

### 2.4. Statistical Analysis

In assessing model fitness, negative binomial, Poisson, and zero-inflated Poisson were explored. Negative binomial regression was found to produce the best model fit, in terms of AIC (8294) and log-likelihood (−8237.99), which is in alignment with findings in similar studies [7]. In this study, the response (i.e., dependent) variables, MR or HB violations, were overdispersed, with large ranges and variances in outcomes, due to most CWSs having zero violations with a mean violation value driven up by a smaller number of more extreme violators, especially for MR violations. When the response variable is overdispersed, negative binomial regression may be a more appropriate model selection [23]. Least absolute shrinkage and selection operator (LASSO) regression was used to improve the choice of variables included in the analysis. Results of LASSO regression showed that for the MR model, %Asian, %Black, %Hispanic or Latino, and source terms did not improve fit, and for the HB model, size terms did not improve fit. For this reason, these variables were not included in each respective final model. Both models were evaluated with and without the inclusion of interaction terms. Any term that was statistically significant in a model that included interaction was also significant when evaluated without an interaction. Odds ratios (ORs) and 95% confidence intervals (CIs) describing the likelihood of MR or HB violations for a CWS were determined via negative binomial regression in R studio version 3.4.4 (R Foundation of Statistical Computing, 2017) with a *p*-value of less than 0.05 determining statistical significance (i.e., the 95% CI does not include 1). Potential predictive variables included the following: %Hispanic or Latino ethnicity, %race (American Indian and Alaska Native; Asian; Black; Native Hawaiian and other Pacific Islander), %population 65 years of age and older, %homeownership, the interaction of %homeownership and %race/ethnicity, RUCA code, system size, source type, owner, and utility year. Percent white was excluded from analysis due to extreme co-correlation, which is to be expected (e.g., high %white is logically associated with low %minority and vice versa).

## 3. Results

### 3.1. Monitoring and Reporting Violations

MR were also negatively associated with medium-sized CWSs (OR = 0.314, 95% CI = 0.148, 0.665) that serve 3301–10,000 people when compared with very small CWSs that serve less than 500 people. Privately owned systems resulted in increased odds (OR = 1.899, 95% CI = 1.455, 2.478), corresponding to an elevated likelihood of incurring a MR violation by 90%, when compared with publicly owned systems. There were no significant findings related to RUCA category for MR violations. Over the study period, MR violations were negatively associated with violation year (OR = 0.928, 95% CI = 0.895, 0.963).

The interaction of %Native Hawaiian (HI) and other Pacific Islanders and %homeownership was significantly linked to MR violations (negative binomial and LASSO regression model: OR = 1.214, 95% CI = 1.013, 1.455; Table 1). The significant interaction between %homeownership and %Native HI and other Pacific Islander revealed that a higher proportion of each is associated with increased odds of a MR violation. However, it is critical to note the large CI associated with this finding, which reflects larger uncertainty.

### 3.2. Health-Based Violations

The only significant demographic predictor of HB violations was %Black (Negative binomial and LASSO regression model: OR = 1.031, 95% CI = 1.018, 1.045; Table 1). With all else constant, an OR of 1.031 indicated that for a 1% increase in %Black within a service area, the odds of incurring a heath-based violation increase by 3%. There were no statistically significant findings related to RUCA category, source water type, owner, and HB violations.

## 4. Discussion

Consistent with the author’s previous work [15], MR violations were 90% more likely in privately owned Virginia CWSs. Previous national studies evaluating differential SDWA compliance by owner revealed that publicly owned utilities incur more HB violations [3,24,25], while privately owned PWSs incur increased MR violations, though some of these efforts have excluded very small-sized utilities from their analysis (Table S3). In the most recent 2006 EPA survey, privately owned (including ancillary) systems made up 49.9% of national CWSs [26] compared with 53.3% of Virginia’s CWSs (Table S2). It is worth noting that while the 662 geocoded CWS study set examined here represents over 75% of Virginians served by municipal water, it contains a lower proportion of private systems than the national and state level (35.6%; Table S2), and they are more likely to be very small-sized (Table S4). By excluding certain system sizes, previous studies may have been unable to uncover relevant trends for end-users of these very small CWSs. This finding may suggest that ownership plays an increasingly critical role in compliance as system size decreases. In fact, from 2000 to 2006, the number of privately owned, very small CWSs nationally that were operating at a loss (i.e., system expenses exceeded revenue) increased from 39 to 52% [26], most likely a critical factor in the MR violations exhibited in these systems. As hypothesized by Fu et al. [24], in an effort to delay or avoid additional expenses caused by HB violations (such as fines, more frequent sampling schedules, or treatment technology updates), privately owned utilities may choose to incur increased MR violations, and this may be even more common in very small-sized CWS. In this study, medium-sized CWSs were 69% less likely to incur MR violations than very small-sized systems. With significant technical, managerial, and financial capacity, larger-sized CWSs are generally more compliant with SDWA regulations [27,28]. Similar trends are seen nationally: as system size increases, CWSs have significantly less MR violations [16,27]. In this study, system size did not impact the likelihood of HB violations, which is also consistent with prior literature [16,29]. It is critical to recognize however, that the binary public/private distinction examined in this study and previous work does not examine the impacts of finer institutional differences in governance on water quality violations [30].

Violation likelihood was not impacted by source water type in the present analysis, though groundwater-sourced large and very large PWSs have been associated with significantly less SDWA violations, compared with other sources, in previous studies [7]. RUCA categories also did not have a significant impact on violation likelihood. However, in recent national analyses, CWSs in less-urbanized areas were found to have a greater likelihood for HB total coliform violations [4], while CWSs in urban independent cities were more likely to experience elevated nitrate levels [6]. It is worth noting that the high number of MR violations across Virginia may be impeding identification of HB water quality issues that exist within the Commonwealth.

Interestingly, the proportion of Black Americans in a CWS service area was positively associated with HB violations: with all else constant, a one-unit increase in %Black within a service area increased odds of a HB violation by 3%. Results also revealed a significant interaction between Native Hawaiian and other Pacific Islanders and homeownership: zip codes with higher proportions of this racial demographic and homeownership resulted in increased odds of MR violations. This finding is seemingly counterintuitive, given that increased wealth, evidenced by homeownership, might be expected to predict lower violation likelihood, which would limit exposure to drinking water contaminants. However, it is critical to emphasize that this demographic group in Virginia accounted for 0.04% of the Virginia population in 2000 [19], resulting in fewer than 1% increases in MR violations across the state. Therefore, it is possible that this finding is a statistical artifact, though further community-scale examinations would be needed to confirm.

Potential interactions between measures of wealth and demographics in relation to water quality compliance are worthy of further investigation. Complex relationships are likely given known historical practices, including redlining, which intentionally denied investment in infrastructure and other community services to areas with higher proportions of Black, Hispanic, or immigrant populations [31]. To date, only two studies [3,7] have assessed the interaction of an SES-based factor and race or ethnicity, both at the national level and excluding very small systems. In both cases, the likelihood of a HB violation increased in CWSs serving low-income, minority populations [3], and in PWSs serving higher proportions of Black and Hispanic populations living below the poverty line [7]. It is worth noting that these studies utilized the county and independent city scale as well as different SES approximations of wealth—median household income, education level, and population below the poverty line—rather than homeownership. When analyzed without assessing potential interactions, higher proportions of homeownership were found to lessen HB arsenic violations in San Joaquin, California CWSs [5]. Although violation likelihood was not influenced by other demographic factors in this study, prior research has shown positive associations nationally with uninsured populations [4]. Beyond SDWA violations, nitrate concentrations have been positively associated with Hispanic/Latino populations [6,8], and negatively associated with %homeownership, %poverty, and %Black populations at the county and independent city scale [6]. Given that this study is unique in including a broad set of demographic predictors and the varied results emerging from previous national studies, further research at the zip code scale in more demographically diverse areas is encouraged.

It is critical to recognize, that given the lack of comprehensive standardized data describing national water service [32], the assumptions used in this work present potential limitations and sources of bias. Most critically, only a subset of VA CWSs could be geocoded, and service areas were delineated based on proximity alone. These assumptions therefore do not capture any deliberate historical exclusion of racial/ethnic minority groups from centralized drinking water access, which has been recognized as an issue in multiple US states [31,33,34,35]. In order to match available sets of RUCA and demographic data, this work relied on data from the 2000 Census, which is two decades out of date. Future research should also examine the impacts of consecutive systems, e.g., wholesalers.

Despite these significant limitations, it is worth emphasizing that this effort begins to explore connections between demographics and drinking water violations where the publicly available data allow. Analyzing demographics and CWS violations at the zip code level affords a finer spatial resolution than merging data at the county scale. The majority of US states do not have CWS service boundaries publicly available, and the limitations of available water quality data are well known [32]. Understanding potential relationships between demographics and home water quality is an urgent need: a recent national survey indicated that the majority of Black Americans do not believe their drinking water is safe to consume [36]. In this study, the demonstration of significant statistical differences based on demographics even given limitations that potentially do not account for intentional exclusion from service is quite notable. Continuing improvements in publicly available service area delineations are necessary to provide even finer scale confirmation of trends. The inclusion of very small-sized systems that serve fewer than 500 people also addresses a critical gap in the literature, as a lack of data availability has forced many previous examinations to exclude these from study.

This work supports mounting evidence that racial/ethnic and SES disparities still exist in access to and quality of safe drinking water, even in high-income nations [3,4,5,6,7,8,13]. Although this work does indicate that annual MR violations have decreased in Virginia between 2006 and 2016, it is critical to ensure that specific populations are not overlooked. There is a need for intersectional investigation of the social determinants of safe drinking water access and quality, as has been proposed in nations such as Canada [37], that can identify potential environmental justice impacts on available SDWA resources and compliance in the United States. With the methodology developed herein, researchers could begin to verify if national trends are masking sub-county-level differences and advocate for standard nationalized efforts to delineate water service, akin to CalEnviroScreen [17]. To date, EPA relies on and makes public a national environmental justice mapping and screening tool (EJSCREEN; [38]) for assessment of combined environmental and demographic indicators, which notably lacks information on drinking water. The cumulative lifetime cancer risk of the population served by Virginia CWSs has been estimated at 3 × 10^−4^, or 3 cases in every 10,000 people, in a 2019 national analysis [39]. Given the demographic and system differences in CWS violations in the subset of VA systems studied, it is important to determine whether these health risks also disproportionately impact similar populations. It is worth noting that the anticipated requirements of the upcoming Revised Lead and Copper Rule for PWS in the US include identifying and publicizing the location of lead service lines. This effort may render the creation of national, standardized service area maps at a fine scale more feasible, which would aid in future explorations of drinking water access and adverse exposures. Until official service line extents are available, the geospatial strategy discussed here offers a strategy for water quality and public health managers to identify those communities most likely at risk.

## 5. Conclusions

In this study, service areas for 662 geocoded Virginia CWSs were delineated at the zip code scale to estimate the demographics of service. Using publicly available data, associations with system characteristics, rural–urban areas, community demographics, and SDWA violations (2006–2016) were investigated through negative binomial regression. Results revealed that HB violations were positively associated with Black Americans: 3% more likely for every one-unit-increase in this predictor. Therefore, in keeping with previous work, our results suggest that in the target area, there is a differentiated access to safe water, mediated by the racial group to which one belongs. This argues for more fine-grained studies of potential problematic systems in minority neighborhoods to determine whether these broad findings are accurate to community experience, and, if so, to identify both engineering and potential sociopolitical issues resulting in differential exposure to adverse drinking water quality events. Findings also reveal that MR violations were 90% more likely in privately owned CWSs and 69% less likely in medium-sized CWSs. Future research should expand intersectional investigations on the social determinants of CWS SDWA compliance and associated environmental health exposures via household taps.

## Figures and Tables

**Table 1:** Demographic and system factors associated with monitoring and reporting and health-based violations in the geocoded subset of Virginia community water systems from 2006–2016 based on negative binomial regression (significance *p* < 0.05).

Factor	Monitoring and Reporting, OR (95% CI)	Health-Based, OR (95% CI)
*Individual Demographics*		
%American Indian or Alaska Native	0.946 (0.797, 1.122)	0.970 (0.766, 1.228)
%Asian	-	0.667 (0.401, 1.110)
%Black	-	1.031 (1.018, 1.045)
%Hispanic or Latino	-	-
%Native Hawaii or Pacific Islander	0.005 (3.270 × 10^−9^, 7.54 × 10^3^)	6.784 × 10^−4^ (1.083 × 10^−11^, 4.249 × 10^4^)
%Homeownership	1.007 (0.990, 1.024)	0.996 (0.966, 1.026)
%65 years of age and older	1.018 (0.990, 1.046)	0.940 (0.880, 1.004)
Interaction of %Homeownership with:		
%Native Hawaii or Pacific Islander	1.214 (1.013, 1.455)	1.198 (0.939, 1.528)
*RUCA Code (Reference: Urban Core)*	-	-
Urban	1.345 (0.876, 2.063)	0.807 (0.345, 1.888)
Large Town Core	1.364 (0.772, 2.411)	1.352 (0.479, 3.811)
Large Town	0.221 (0.045, 1.078)	2.144 (0.681, 6.747)
Small Town Core	1.340 (0.825, 2.176)	0.945 (0.395, 2.262)
Small Town	1.264 (0.651, 2.455)	1.117 (0.371, 3.367)
Isolated Rural Area	1.089 (0.709, 1.672)	0.757 (0.327, 1.750)
*Size (Reference: Very Small)*		
Small	1.048 (0.786, 1.397)	-
Medium	0.314 (0.148, 0.665)	-
Large	0.488 (0.174, 1.365)	-
Very Large	0.132 (0.010, 1.703)	-
*Source (Reference: Groundwater)*		
Surface Water	-	1.101 (0.603, 2.011)
Groundwater Under the Influence of Surface Water	-	1.087 (0.336, 3.520)
*Operation*		
Private (Reference: Public)	1.899 (1.455, 2.478)	1.425 (0.837, 2.428)
*Year*	0.928 (0.895, 0.963)	0.972 (0.910, 1.039)

Note: Variables not included in the final respective regression model, based on the outcome of least absolute shrinkage and selection operator (LASSO) regression, are indicated by a “-”. CI = confidence interval, OR = odds ratio. RUCA = Rural urban commuting area from the US Department of Agriculture.

## Data Availability

Publicly available datasets were analyzed in this study. This data can be found here: https://www.epa.gov/ground-water-and-drinking-water/safe-drinking-water-information-system-sdwis-federal-reporting (accessed on 26 October 2021).

## Supplementary Materials

The following are available online at https://www.mdpi.com/article/10.3390/ijerph182413254/s1, Table S1: Summary of peer-reviewed studies that analyze public or community water system (P/CWS) violations; Table S2: Descriptive statistics of demographic factors for Virginia zip codes; Table S3: Descriptive statistics of community water systems included in the study subset (*n* = 662) compared with all of Virginia (*n* = 1133); Table S4: Percentage of private and public community water systems by size for the study subset (*n* = 662) compared with all of Virginia (*n* = 1133); Figure S1: Visualization of community water system service area delineation at the zip code level.
