# Supplementary material for: Approximating Community Water System Service Areas to Explore the Demographics of SDWA Compliance in Virginia
Source: Int J Environ Res Public Health. 2021 Dec 16;18(24):13254. doi: 10.3390/ijerph182413254 (PMC8706897; doi:10.3390/ijerph182413254)
Supplement: Supplementary file 1 [file ijerph-18-13254-s001.zip › ijerph-1459885-supplementary.pdf]

SUPPLEMENTARY MATERIAL

Table S1: Summary of peer-reviewed studies that analyze public or community water system (P/CWS) violations or contaminant concentrations in association with at least one demographic variable, using a geographic unit of county/independent city or smaller.

Author	Scale	Analytical Method	Demographic and Health Variables	Primary finding(s) related to demographics
Cory and Rahman (2009)[7]	Place: Arizona<br>Systems: PWS<br>Violations: As MCL<br>Geography: Zip code<br>Time: 1999-2004	Binary logistic regression	%Black; %Hispanic; %Minority [Black & Hispanic]; Income per capita; Avg. income per household; Avg. value of house; Persons per household; Arsenic >10 ppb	•No evidence that minority and low-income populations were disproportionately served by CWSs with arsenic violations
Balazs et al. (2011)[5]	Place: San Joaquin, CA<br>Systems: CWS<br>Contaminant: Nitrate<br>Geography: Block Group<br>Time: 1999-2001	Linear regression (size stratified)	%Latino; %Non-Latino people of color; %Homeowners; Nitrate concentration	•Among smaller systems, every 1% Latino was associated with an estimated increase of 0.44 mg/L of nitrate
Balazs et al. (2012)[3]	Place: San Joaquin, CA<br>Systems: CWS<br>Violations: Arsenic MCL<br>Geography: Block Group<br>Time: 2005-2007	Linear regression (size stratified) & fisher's exact tests	%People of color; %Homeowners; Avg. arsenic concentration; Note: arsenic MCL used for Fisher	•CWSs with higher rates of homeownership had lower odds of receiving an MCL violation; those serving higher percentages of minorities had higher odds of an MCL violation<br>•Higher homeownership rate was associated with lower arsenic levels, with the relationship strengthen in smaller systems
Stillo & MacDonald Gibson (2017)[11]	Place: Wake County, NC<br>Systems: CWS vs. Wells<br>Violations: Total Coliform & *E. coli* MCLs<br>Geography: County<br>Time: Wells 2014; CWS 2009-2013	Population intervention model	County population; Geographic region; Population in poverty; County's uninsured rate above the NC median; Emergency department in county; County visits to emergency department for acute gastrointestinal issues; Population exposed to microbiological violations in CWSs or comparable quality in wells monthly	•The model resulted in 25 emergency department visits per year that could be avoided if communities served by private wells received drinking water quality comparable to that in Wake County community water systems.<br>•The risk of visiting an emergency department for acute gastrointestinal issues is 22% higher in under-bounded communities (served by private wells) than in areas with community water system service.
Switzer and Teodoro (2017)[4]	Place: National<br>Systems: PWS (size L-VL)<br>Violations: All MCL and TT<br>Geography: County or Independent City<br>Time: 2010-2013	Negative binomial regression	%Hispanic; %Black; %High school educated; %Bachelor's degree; %Below the poverty line; Median household income; Interaction of %below poverty line & race/ethnicity measures; MCL & TT count	•Race and ethnicity have a major impact on the number of violations committed by a utility, but the relationship is conditional on poverty<br>•%Hispanic & %Black population significantly increases violations when %population below the poverty line is greater than 30%
Allaire et al. (2018)[1]	Place: National<br>Systems: CWS (size S-VL)<br>Violations: All MCL, MRDL, & TT<br>Geography: County<br>Time: 1982-2015	Probit & LASSO regression	%Non-white; Housing Density; Median household income; MCL, MRDL, & TT presence	•Low-income rural areas have a larger compliance gap than higher-income rural areas, that becomes especially pronounced after the new disinfection byproduct rules in the early 2000s<br>•Low-income population is associated with a higher likelihood of total coliform violations
McDonald and Jones (2018)[2]	Place: National<br>Systems: CWS<br>Violations: All types<br>Geography: County<br>Time: 2011-2015	Logistic regression & odds ratios	Non-Hispanic Blacks, Asians, and Whites; Renters; adults with less than a high school education; uninsured households, median income; Initial & repeat violation presence	•Initial and repeat violations are positively associated with the proportion of those who were uninsured. A 1 unit increase in the proportion of uninsured in a county (with all else equal) increased the odds of an initial & repeat violation by 77% & 67%, respectively.

Schaider et al. (2019)[6]	Place: National<br>Systems: CWS (excluding purchased water)<br>Contaminant: Nitrate<br>Geography: County and/or City<br>Time: 2010-2014	Linear regression (mixed-effects)	%Hispanic; %Non-Hispanic Black; %Homeownership; %Families with income below the poverty line; land-use variables	•%Hispanic residents were positively associated with nitrate, while %African American residents were negatively associated with that same contaminant, at both the county and city level<br>•%residents living in poverty and %homeownership were negatively associated with nitrate only at the county and city level, respectively

Note: PWS-public water system; CWS-community water system; MCL-maximum contaminant level; TT-treatment technique; MRDL-maximum residual disinfection level; LASSO-Least absolute shrinkage and selection operator.

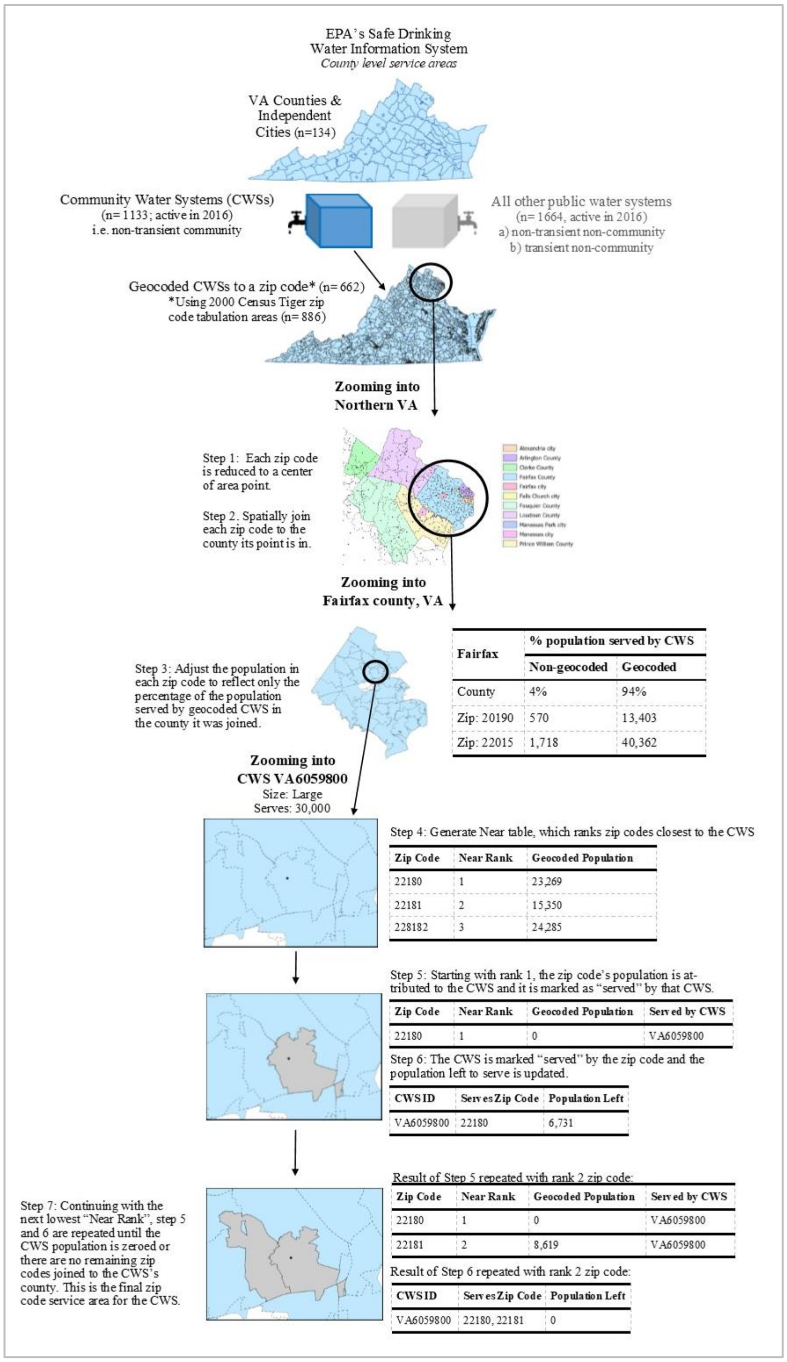

Figure S1: Visualization of community water system service area delineation at the zip code level in ESRI's ArcGIS Pro, including an illustrative example (Fairfax County in Northern Virginia, which with 5 geocoded, active systems based on and a population of 1,081,699 based on the 2000 Census, comprising one of the more complex delineation areas).

Table S2. Descriptive statistics of demographic factors for Virginia zip codes (n=886).

**Demographic Factor**	**% Average (Range)**
% American Indian or Alaska Native	0.43 (0-22.85)
% Asian	2.12 (0-44.23)
% Black	17.67 (0-98.23)
% Hispanic or Latino	2.47 (0-36.76)
% Native Hawaii or Pacific Islander	0.04 (0-1.98)
% White	80.3 (0-100)
% Other Race	0.19 (0-17.2)
% Homeownership	75.06 (0-100)
% 65 years of age and older	13.95 (0-50.41)

Note: Races do not include Hispanic or Latino Ethnicity.

Table S3. Descriptive statistics (% of total) of community water systems included in the study subset (n=662) compared to all of Virginia (n=1,133).

Community Water System Characteristic	Study Subset	Virginia
Size		
Very Small	55.74	64.82
Small	28.70	22.23
Medium	9.37	6.96
Large	4.38	4.55
Very Large	1.81	1.43
Source		
Groundwater	57.10	67.50
Surface Water	35.35	27.41
Groundwater Under the Influence of Surface Water	7.55	5.09
Owner		
Public	64.65	46.96
Private	35.35	53.04
Rural; Urban Commuting Area		
Urban Core	15.56	-
Urban	24.32	-
Large Town Core	5.59	-
Large Town	3.02	-
Small Town Core	11.78	-
Small Town	5.29	-
Isolated Rural Area	34.44	-
Demographics		
American Indian or Alaska Native	0.30	0.31
Asian	0.63	3.76
Black	15.46	20.05
Hispanic or Latino	1.69	4.66
Native Hawaii or Pacific Islander	0.02	0.06
Homeownership	76.26	68.09
65 years of age and older	14.65	11.19

Note: Community water system characteristics are for 2016 from the Environmental Protection Agency's Safe Drinking Water Information System. "-" indicates unknown, as all systems in Virginia were not able to be geocoded; Using 2000 Census demographics.

Table S4: Percentage of private and public community water systems by size for the study subset (n=662) compared to all of Virginia (n=1,133).

**System Size**	**Study Subset**		**Virginia**	
	Private	Public	Private	Public
Very Small	30.36	25.38	46.19	18.50
Small	4.38	24.32	5.58	16.46
Medium	0.15	9.21	0.27	6.81
Large	0.3	4.08	0.44	4.34
Very Large	0.15	1.66	0.09	1.33

Note: Community water system characteristics are for 2016 from the Environmental Protection Agency’s Safe Drinking Water Information System.
